# *Bacillus coagulans* prevents the decline in average daily feed intake in young piglets infected with enterotoxigenic *Escherichia coli* K88 by reducing intestinal injury and regulating the gut microbiota

**DOI:** 10.3389/fcimb.2023.1284166

**Published:** 2023-11-14

**Authors:** Yanyan Zhang, Xiaorong Tian, Yi Dong, Rui Li, Meng Shen, Dan Yi, Tao Wu, Lei Wang, Di Zhao, Yongqing Hou

**Affiliations:** Hubei Key Laboratory of Animal Nutrition and Feed Science, Engineering Research Center of Feed Protein Resources on Agricultural By-products, Ministry of Education, Wuhan Polytechnic University, Wuhan, China

**Keywords:** piglets, *Bacillus coagulans*, enterotoxigenic escherichia coli, average daily feed intake, intestine, gut microbiota

## Abstract

**Background:**

Enterotoxigenic *Escherichia coli* (ETEC), an important intestinal pathogen, poses a significant threat to the intestinal health of piglets. *Bacillus coagulans* (BC), a potential feed additive, can improve the intestinal function of piglets. However, the effects of BC on growth performance and intestinal function in ETEC-infected piglets are still unclear. In this study, 24 7-day-old piglets were randomly assigned to three treatment groups: control group (fed a basal diet), ETEC group (fed a basal diet and challenged with ETEC K88) and BC+ETEC group (fed a basal diet, orally administered BC, challenged with ETEC K88). During Days 1-6 of the trial, piglets in the BC+ETEC group were orally administered BC (1×10^8^CFU/kg). On Day 5 of the trial, piglets in the ETEC and BC+ETEC groups were orally administered ETEC K88 (5×10^9^CFU/piglet). Blood, intestinal tissue, and content samples were collected from the piglets on Day 7 of the trial.

**Results:**

The average daily feed intake in the ETEC group was significantly reduced compared to that of the control group. Further research revealed that ETEC infection significantly damaged the structure of the small intestine. Compared to the control group, the villus height and surface area of the jejunum, the ratio of villus height to crypt depth in the duodenum and jejunum, and the activities of catalase and total superoxide dismutase in the jejunum were significantly reduced. Additionally, the levels of myeloperoxidase in the jejunum, malondialdehyde in the plasma and jejunum, and intestinal epithelial apoptosis were significantly increased in the ETEC group. However, BC supplementation had significantly mitigated these negative effects in the BC+ETEC group by Day 7 of the trial. Moreover, BC supplementation improved the gut microbiota imbalance by reversing the decreased numbers of *Enterococcus*, *Clostridium* and *Lactobacillus* in jejunum and *Escherichia coli*, *Bifidobacterium* and *Lactobacillus* in the colon, as well as the increased number of *Escherichia coli* in the jejunum induced by ETEC K88.

**Conclusions:**

Overall, BC supplementation reduced the decline in average daily feed intake in ETEC K88-infected piglets by attenuating intestinal epithelial apoptosis and oxidative stress and regulating the gut microbiota. This suggests that BC may be used to prevent intestinal infections caused by ETEC in piglets.

## Introduction

The intestine is the largest immune organ and primarily made up of the mucous layer, epithelial cells, immune cells in the lamina propria and tight junctions (Modina et al., 2021). The epithelial barrier function and the mucosal immune system play important roles in protecting the intestinal tract of piglets from damage (Pluske et al., 2018; Zhang et al., 2022). The intestinal mucosal system is the first line of defense against bacteria and harmful substances entering the body by the adaptive immune system (lymphocytes) and the innate immune system (dendritic cells, macrophages and cytokines) (Pluske et al., 2018; Peng et al., 2021). The intestinal epithelial cells, such as immune sentinel cells, play important immunomodulatory properties by recognising pathogenic microorganisms and secreting growth factors and interleukins (Moeser et al., 2017). Additionally, the intestine of suckling piglets is the main site of nutrient digestion and absorption (Wang et al., 2018). Therefore, it is of great significance for piglet breeding to maintain the intestinal health of piglets.

Although the intestinal structure and function of piglets at birth are still not fully developed (Li et al., 2022), the digestive organs of newborn piglets grow rapidly after birth, particularly in the absorption area of the small intestine (Sangild et al., 2013). Previous studies have reported that in mammals, nutrient digestion and absorption mainly occur in the small intestine in mammals (Mößeler et al., 2016). Therefore, the small intestinal morphology of piglets plays a crucial role in the digestion and absorption of nutrients. As piglets grow, the shape and structure of the small intestine undergo changes and tend to mature, such as an increase in villus height, crypt depth, and intestinal wall thickness (Hoyles and Wallace, 2010; Chen et al., 2016). The intestinal epithelial cells originate from stem cells in the crypt and differentiate as they migrate to the top of the villus. The lifespan of intestinal epithelial cells in piglets is short; typically, they only survive for 2 to 5 days and are then subject to apoptosis. During upward migration, they continually enhance the structure and function of the piglets’ intestine, increase the activity of digestive enzymes and impact the ratio of villus height to crypt depth (V/C), which is directly related to the renewal of intestinal epithelial cells (Fan et al., 1997; Ferraris, 2001; Mößeler et al., 2016). Therefore, the dynamic balance between the proliferation and apoptosis of intestinal epithelial cells is crucial for maintaining intestinal mucosal integrity.

Furthermore, the intestinal microflora plays a significant role in the development of the immune system and the overall health of piglets (Guevarra et al., 2019). The intestinal barrier of piglets gradually develops and matures after birth, mainly influenced by the colonisation and development of the early intestinal microbiota as well as maternal nutrition and other factors. The early colonisation of the intestinal microbiota has a substantial impact on the development of intestinal structure and function. Sow faeces and breast milk microbiota are the primary sources of intestinal microbiota for newborn piglets (Mach et al., 2015; Chen et al., 2018; Ganal-Vonarburg et al., 2020). The rapidly growing intestinal microbiota in newborn piglets has a significant interaction with intestinal morphology, structure, barrier function and the mucosal immune system to promote long-term symbiosis (Hasan et al., 2018; Beaumont et al., 2020; Ganal-Vonarburg et al., 2020). Therefore, the changes in the intestinal morphology of piglets after birth are primarily influenced by the gut microbiota (Wiyaporn et al., 2013).

However, certain pathogens hinder the healthy development of the intestine in young piglets, particularly enterotoxin-producing *Escherichia coli* (ETEC), which negatively affects the intestinal structure and reduces the growth performance of young piglets, resulting in significant economic losses to the pig industry. The ETEC belongs to the Gram-negative bacteria and is a member of the *Escherichia* genus of Enterobacteriaceae. The main route of ETEC infection is through faecal-oral transmission. Newborn piglets are easily exposed to ETEC pathogens when they ingest breast milk or move in the enclosure as these pathogens are discharged from the diarrhoea of animals in the environment and can survive for at least 6 months when protected by faeces (Gyles et al., 2010). After infecting the host, ETEC must pass through the stomach before colonising the small intestine. The acidity of the adult pig’s stomach is extremely strong, which acts as an important defense against the invasion of *Escherichia coli* (*E. coli*) and other microorganisms. In comparison, piglets have a weaker acidity of their stomachs and fewer digestive enzymes in the stomach and duodenum, making it easier for ETEC to survive (Xia et al., 2018). Once the bacteria reach the small intestine, they can use adhesins (such as K88, K99, 987p, F41, F42, among others) to attach to the intestinal mucus layer or epithelial cells where they propagate rapidly, reaching 10^9^ CFU/g in the jejunum and ileum (Mainil et al., 1990; Gyles et al., 2010). Subsequently, ETEC disrupts the balance of water and electrolytes in intestinal epithelial cells by releasing enterotoxins (ST and LT), resulting in watery diarrhoea (Nakanishi et al., 2006). The ETEC-induced intestinal infection in piglets has caused significant economic losses in the breeding industry (Zhang et al., 2018). Therefore, it is urgent to develop nutritional regulators for the prevention and treatment of ETEC-induced intestinal infection in piglets.

*Bacillus coagulans* (BC), as a facultative anaerobic, Gram-positive and non-pathogenic bacterium, can form spores and produce lactic acid (Özüsa˘ glam, 2010). Spore-forming BC exhibits strong resistance to extreme environmental conditions, which allows it to adapt to the acidic and hypoxic environment of the gastrointestinal tract and survive in the intestine, where is exerts the functions of lactic acid bacteria (Hung et al., 2012). Previous studies have demonstrated that BC increased the feed conversion ratio, enhanced intestinal antioxidant capacity and immune function, regulated gut microbiota balance and reduced heavy metal toxicity (Zhou et al., 2020). Moreover, as a probiotic dietary supplement, BC is widely used to maintain intestinal health in humans and animals (Endres et al., 2011). Previous studies reported that BC showed positive outcomes in piglets, poultry production and aquaculture (Zhou et al., 2020). It also improved piglet growth performance by reducing the incidence of diarrhoea, enhanced the growth of broiler chickens by promoting a balanced intestinal microbiota and increased the final weight of shrimp and grass carp (Cavazzoni, 1998; Wang and Gu, 2010; Wang, 2011; Hung et al., 2012; Wu et al., 2018). However, it remains unclear whether *B. coagulans* can improve the intestinal damage caused by ETEC infection in piglets. Therefore, this study investigated the effects of *B. coagulans* on growth performance and intestinal function in ETEC K88-infected piglets, as well as the underlying mechanisms.

## Materials and methods

### Animals and experimental design

The pig experiment was approved by the Institutional Animal Care and Use Committee and was conducted according to the Guide for the Care and Use of Laboratory Animals at Wuhan Polytechnic University (approval code WPU201910001). Twenty-four healthy 7-day-old crossbred piglets (Duroc × Landrace × Yorkshire), including 12 females and 12 males, were purchased from commercial pig farms. All piglets had a similar body weight of 1.803 ± 0.049 kg and were randomly assigned into three groups with eight replicates each (1 piglet per replicate): the Control group, the ETEC group and the BC+ETEC group. The piglets were housed in stainless-steel metabolic cages (1.0 × 1.5 m², one piglet per cage), and the ambient temperature was maintained at 29-34°C. They were fed a milk substitute as a basal diet, which was purchased from Shanghai Gaode Feed LTD. After 3 days of adaptation, the piglets in the BC+ETEC group were orally administered BC (1 × 10^8^ CFU/kg body weight, 5 × 10^7^ CFU BC per 1 mL PBS) at 5:00 every day from Days 1-6 of the trial. The piglets in the control and ETEC groups were orally administered the same volume of PBS. Just on Day 5 of the trial, each piglet in the ETEC and BC+ETEC groups was orally administered 2 mL PBS with ETEC K88 (2.5 × 10^9^ CFU/mL) twice, while the piglet in the control group was orally administered 2 mL PBS. Feed intake (on Days 1-6 of the trial) and body weight (only on Days 1 and 7) were recorded to analyse the growth performance of the piglets. The average daily feed intake (ADFI) (g/day) was calculated as the feed intake (g, eight piglets per treatment group on Days 1-6 of the trial) divided by 2. To ensure consistency in the dosage of each administration, the ETEC K88 and BC inoculums were prepared fresh each day. The ETEC K88 strain was cultured in Luria-Bertani (LB) medium at 37°C. After 9 hours of growth, the ETEC K88 strain (OD_600_ = 0.9) was collected by centrifugation at 6,000 g for 10 minutes and suspended in PBS (2.5 × 10^9^ CFU/mL). The BC strain was cultured in MRS medium at 37°C. After 24 hours of growth, the BC strain (OD_600_ = 2.5) was collected by centrifugation at 7,000 *g* for 10 minutes and suspended in PBS (5 × 10^7^ CFU/mL). Pig faeces were classified into four levels: 0, normal; 1, soft faeces; 2, mild diarrhoea; and 3, severe diarrhoea (Bhandari et al., 2008). On Day 7 of the trial, pentobarbital sodium (50 mg/kg BW) was used to anesthetize the piglets by intravenous injection. Subsequently, samples of the intestinal tissue (jejunum, ileum and colon), mucosa and content were collected from the dissected piglets. Five-cm intestinal segments were obtained, washed with precooled PBS and fixed with 4% paraformaldehyde for morphological measurements. Other samples of intestinal tissue, mucosa, and content were rapidly frozen in liquid nitrogen and stored at -80°C until analysis.

### The measurement of blood biochemical and heamatological parameters

On Day 7 of the trial, all piglets were orally administered D-xylose at 7:00 AM. After 1 hour, blood samples were obtained from the anterior vena cava of the piglets using 10-mL anticoagulant and non-anticoagulant vacuum tubes. The non-anticoagulant vacuum tubes with blood were placed at 37°C for 30 minutes. The plasma and serum were then separated by centrifuging at 3,000 rpm for 15 minutes, and then serum was used for the determination of D-xylose and biochemical parameters, including total serum bilirubin (TB), total protein (TP), albuminuria (ALB), aspartate transaminase (AST), alanine transaminase (ALT), total serum cholesterol (TC), triglyceride (TG), glucose (GLU), total serum calcium (TC), progesterone (p), creatinine (Crea), high-density lipoprotein cholesterol (HDL), low-density lipoprotein cholesterol (LDL), glutamyltransferase (GGT), blood urea nitrogen (BUN) and creatine kinase (CK). These serum biochemical parameters were measured using commercial kits purchased from Hitachi Hi Tech Co., Ltd, with a Hi-tachi 7020 Automatic Biochemical Analyzer (Hitachi, Tokyo, Japan). The whole blood in the anticoagulant vacuum tubes was used to measure haematological parameters. An automatic blood analyser (ADVIA^®^ 2120/2120i, Siemens Healthcare Diagnostics Inc.) was used for haematological parameter measurements following the operational instructions of the manufacturer.

### Intestinal morphology measurement

The 5-cm segments of the duodenum, jejunum and ileum fixed in 4% paraformaldehyde were used to measure the morphology of the intestines. The collected segments were 5-10 cm from the front of the duodenum, the front of the middle 15 cm of the jejunum and the front of the middle 15 cm of the ileum, respectively. Subsequently, the paraformaldehyde-fixed intestinal segments were sent to Wuhan Bode Biological Engineering Co., Ltd. for further analysis. Intestinal morphology was determined using a light microscope (Leica, Solms, Germany). A linear ocular micrometre with a computer-assisted morphometric system (Leica, Solms, Germany) was employed to measure the vertically oriented villus height from the villus tip to the crypt mouth, the vertically oriented crypt depth from the crypt mouth to the base, and the villus width. Villus surface area was determined by multiplying villus height by villus width.

### Redox status measurement

The mucosa and plasma were used to measure the redox status. The plasma samples were collected following the method described above. The mucosa samples were scraped from the inner lining of the longitudinally cut intestine wrapped in tin foil and stored at -80°C until further analysis. For measuring the intestinal redox status, the ground mucosa samples were thoroughly mixed with nine times the volume of pre-cooled PBS. The supernatants were then obtained by centrifuging at 3,500 rpm for 15 minutes at 4°C, and used for measuring the activities of catalase (CAT), total superoxide dismutase (T-SOD), glutathione peroxidase (GSH-Px) and myeloperoxidase (MPO) as well as the levels of hydrogen peroxide (H_2_O_2_) and malondialdehyde (MDA). These indicators were measured using commercially available kits from Nanjing Jiancheng Bioengineering Institute, Nanjing, China, including the CAT assay kit (ultraviolet), the T-SOD assay kit (hydroxylamine method), the GSH-PX assay kit (colorimetric method), the total myeloperoxidase assay kit, the H_2_O_2_ assay kit and the MDA assay kit (TBA method). The same commercially available kits were also used to determine these indicators in the plasma samples.

### Assessment of apoptosis in the ileum epithelium

To assess the impact of BC supplementation on the apoptosis level of ileum epithelium in piglets infected with ETEC, we used the TUNEL Apoptosis Detection Kit (FITC, MK1027) purchased from BOSTER Biotechnology in this study. The ileum samples fixed in 4% paraformaldehyde were embedded in paraffin and then routinely dewaxed into water. The specimen was treated with 0.01M TBS 1:200, digested with Proteinase K at 37°C for 10-15 minutes and washed with TBS three times for 2 minutes each time. To keep the slices moist, 20 μL of labelling buffer per tablet was added. For each slice, 1 μL of TdT and 1 μL of DIG-d-UTP were added to 18 μL of labelling buffer and thoroughly mixed. The excess liquid on the slice was removed and added to the marking liquid (20 μL per tablet). The sample was placed in a wet box and marked at 37°C for 2 hours. The labelled slices were washed with 0.01M TBS three times for 2 minutes each time. Each slice was then spiked with sealing liquid (50 μL) and kept at room temperature for 30 minutes, after which the sealing solution was removed by shaking. The biotinylated anti-digoxin antibody was diluted with SABC diluent (1:100), and 50 μL per piece was added to the specimen. Subsequently, the sample was placed in a wet box and incubated at 37°C for 30 minutes, followed by washing with 0.01M TBS three times for 2 minutes each time. The SABC was diluted (1:100) with SABC diluent, and 50 μL per tablet was added to the slice for a 30-minute incubation at 37°C. The slice was then washed with 0.01 M TBS four times for 5 minutes each time. If necessary, DAPI dye solution (product No. AR1176, AR1177) was used for slight re-dyeing and washed with distilled water. A fluorescence microscope was employed for observation.

### Determination of the gut microflora in the digesta of jejunum, ileum and colon

The segments of intestine with digesta were taken from the jejunum, ileum and colon and placed on ice, respectively. Subsequently, the digesta in the jejunum (n = 8), ileum (n = 5) and colon (n = 8) were obtained using the method described by Zhang et al. (2022). For the measurement of microorganisms in the jejunum, ileum and colon, the digesta were thoroughly mixed and stored at -80°C until analysis. The DNA was extracted as previously described (Wu et al., 2021). Droplet digital PCR (ddPCR) was employed to determine the numbers of *Escherichia coli*, *Bifidobacterium, Enterococcus, Clostridium, Lactobacillus* and total eubacteria in the jejunum, ileum and colon, following the method described by Wu et al. (2021). A QX200 Droplet Digital PCR system (Bio-Rad) was used, and the primers used in this study are listed in **Table 1**.

**Table 1:** Primers used in this study.

Strain	Forward (5′–3′)	Reverse (5′–3′)
*Escherichia coli*	CATGCCGCGTGTATGAAGAA	CGGGTAACGTCAATGAGCAAA
*Bifidobacterium*	TCGCGTC(C/T)GGTGTGAAAG	CCACATCCAGC(A/G)TCCAC
*Enterococcus*	CCCTTATTGTTAGTTGCCATCATT	ACTCGTTGTACTTCCCATTGT
*Clostridium*	AATGACGGTACCTGACTAA	CTTTGAGTTTCATTCTTGCGAA
*Lactobacillus*	AGCAGTAGGGAATCTTCCA	CACCGCTACACATGGAG
Total eubacteria (16S rRNA)	CGGTCCAGACTCCTACGGG	TTACCGCGGCTGCTGGCAC

### Statistical analysis

One-way analysis of variance (ANOVA) was performed to analyse all data in this study, using the SPSS 17.0 statistical software (SPSS Inc., Chicago, USA). The data are expressed as mean values ± SEM. A *post hoc* analysis using the Tukey’s or Tukey-Kramer’s multiple comparison test, as approriate, was performed to determine the differences among treatment means. The faecal scores were expressed as weighted averages and analysed via an χ^2^ test. A significance level of *P* < 0.05 was adopted.

## Results

### BC supplementation improved the ADFI of young piglets infected with ETEC K88

**Figure 1:**
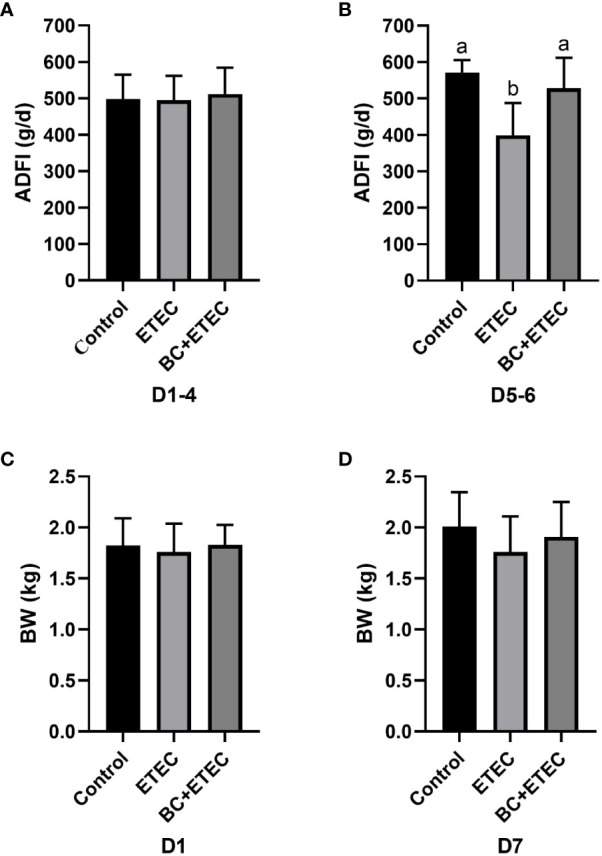
Effects of *B. coagulans* on the growth performance of young piglets infected with ETEC K88. **(A)** The ADFI of piglets in the control, ETEC and BC+ETEC groups before challenge with ETEC K88 (on d1-4). **(B)** ADFI of piglets in the control, ETEC and BC+ETEC groups after challenge with ETEC K88 (on d5-6). **(C)** The BW of piglets in the control, ETEC and BC+ETEC groups on D1. **(D)** The BW of piglets in the control, ETEC and BC+ETEC groups on D7. ADFI, average daily feed intake; BW, body weight; BC, *B. coagulans*; d, day. Data were presented as mean ± SEM (n = 8). ^a,b^ Means followed by different letters above the graphs indicate statistically significant differences (*p* < 0.05) (**Figure 1B**). The alphabetical assignment is not available as *post-hoc* test revealed no significant difference between groups (**Figures 1A, C, D**).

Prior to the ETEC K88 challenge, BC supplementation did not affect the ADFI of young piglets on the Days 1-4 of the trial (**Figure 1A**, *p* = 0.877). Compared to the control group, the ADFI in the ETEC group was significantly reduced on the Days 5-6 of the trial (**Figure 1B**, *P* = 0.022). However, the ADFI in the BC+ETEC group was significantly increased compared to that of the ETEC group on Days 5-6 of the trial, and the ADFI of the BC+ETEC group remained comparable to that of the control group on Days 5-6 of the trial (**Figure 1B**, *P* = 0.022). The BC supplementation prevented the decline in ADFI of ETEC K88-infected young piglets. **Figure 1C** shows that there was no significant difference in the initial BW of young piglets among the three groups (**Figure 1C**, *p* = 0.824). Albeit insignificant, ETEC K88 infection appeared to stunt the growth of piglets as compared to the control group, whereas BC supplementation tended to increase the BW of ETEC K88-infected piglets on Day 7 of the trial (**Figure 1D**, *p* = 0.34). Compared with the control group, the faecal score of piglets in the ETEC group was significantly increased (*p* < 0.05). Compared with the ETEC group, the faecal score of piglets in the BC+ETEC group was not significantly changed, but there was a downward trend (*p* > 0.05) (data no shown).

### BC supplementation improved the blood biochemical and haematological parameters of young piglets infected with ETEC K88

Compared to the control group, ETEC K88 infection significantly decreased the activities of AST and CK as well as the level of BUN while increasing the levels of HDL and GGT in the blood of piglets. In the BC+ETEC group, the level of TG in the blood of piglets was significantly reduced compared to both the control and ETEC groups (**Table 2**). Although BC supplementation did not improve the abnormality in these biochemical indicators in ETEC K88-infected piglets, it also did not exacerbate the adverse effects of ETEC K88 infection on the blood biochemical indicators in piglets. Compared to the control group, ETEC K88 infection significantly reduced the number of WBCs and MONO and increased the levels of CHCM, MCHC and HDW, as well as the numbers of BASO and LUC in the blood of piglets. Further, BC supplementation in the BC+ETEC group significantly reduced the reduction in WBC and MONO counts and the increase in BASO and LUC in the blood of piglets. In addition, compared to the control and ETEC groups, the number of EOS in the blood of piglets in the BC+ETEC group was significantly increased (**Table 3**), and BC supplementation improved the abnormal haematological parameters in piglets caused by ETEC infection. Compared to the control group, ETEC K88 infection decreased the D-xylose content in the blood. Compared to the ETEC group, BC supplementation significantly increased the content of D-xylose in the blood of ETEC K88-infected piglets (**Table 2**).

**Table 2:** Effects of *B. coagulans* on blood biochemical of young piglets infected with ETEC K88.

Items	Control	ETEC	BC+ETEC	*P*
**TB**	0.038 ± 0.052	0.043 ± 0.049	0.086 ± 0.083	0.271
**TP**	54.786 ± 3.202^b^	59.413 ± 6.198^ab^	62.994 ± 8.777^a^	0.060
**ALB**	28.263 ± 3.092	26.838 ± 3.181	28.235 ± 2.051	0.524
**AST**	35.286 ± 5.229^a^	23.714 ± 2.657^b^	23.007 ± 1.732^b^	<0.001
**ALT**	43.51 ± 5.124	39.375 ± 3.815	41.143 ± 6.833	0.327
**ALP**	572.143 ± 86.261	500.673 ± 78.696	586.229 ± 99.563	0.142
**TC**	71.685 ± 11.247^ab^	78.181 ± 4.407^a^	65.687 ± 10.02^b^	0.039
**TG**	42.412 ± 6.176^a^	39.029 ± 3.195^a^	28.726 ± 2.474^b^	<0.001
**GLU**	80.5 ± 11.379	91.629 ± 8.825	80.535 ± 12.541	0.090
**CA**	9.836 ± 0.335	9.89 ± 0.515	10.031 ± 0.537	0.698
**P**	6.139 ± 0.941	6.266 ± 0.593	6.418 ± 0.633	0.754
**CREA**	64.673 ± 4.217^ab^	69.939 ± 5.301^a^	59.046 ± 10.607^b^	0.024
**HDL**	45.666 ± 6.559^b^	55.935 ± 8.414^a^	48.584 ± 7.55^ab^	0.035
**LDL**	49.048 ± 11.476	64.955 ± 26.42	54.089 ± 14.106	0.237
**BUN**	16.425 ± 1.121^a^	13.057 ± 1.688^b^	15.618 ± 2.58^a^	0.005
**GGT**	35.136 ± 5.786^b^	48.77 ± 7.33^a^	52.834 ± 6.012^a^	<0.001
**CK**	267.848 ± 49.54^a^	186.796 ± 21.258^b^	181.918 ± 32.61^b^	<0.001
**D-xylose**	0.437 ± 0.307^ab^	0.31 ± 0.194^b^	0.674 ± 0.256^a^	0.03

TB, total bilirubin; TP, total protein; ALB, serum albumin; AST, aspartate transaminase; ALT, alanine transaminase; TC, Total serum cholesterol; TG, triacylglycerol; GLU, glucose; CA, calcium; P, phosphorus; Crea, creatinine; HDL, high density lipoprotein; LDL, low density lipoprotein; GGT, gamma-glutamyl transferase; BUN, blood urea nitrogen; CK, creatine kinase. Data was presented as mean ± SEM (n = 8). ^a,b,c^ means with different superscript letters are significantly different (p < 0.05).

**Table 3:** Effects of *B. coagulans* on blood hematological parameters of young piglets infected with ETEC K88.

Items	Control	ETEC	BC+ETEC	*P*
**WBC (10e^9^/L)**	8.517 ± 1.21^a^	6.382 ± 0.57^b^	8.861 ± 1.575^a^	0.001
**RBC (10e^12^/L)**	6.373 ± 0.79	6.831 ± 0.738	6.774 ± 0.908	0.484
**HGB (g/dL)**	119.875 ± 8.254	120.429 ± 3.995	118.714 ± 7.814	0.882
**HCT (%)**	40.275 ± 2.81	40.513 ± 3.606	39.271 ± 2.261	0.675
**CH (pg)**	21.163 ± 1.684	20.413 ± 1.184	20.35 ± 1.177	0.433
**MCV (fL)**	63.713 ± 5.345	59.5 ± 3.055	60.538 ± 3.057	0.111
**MCHC (g/dl)**	297.375 ± 3.068^b^	303.571 ± 2.259^a^	302 ± 7.819^ab^	0.057
**MCH (pg)**	18.938 ± 1.543	18.213 ± 1.044	18.275 ± 0.962	0.429
**CHCM (g/dL)**	332.875 ± 4.998^b^	343.625 ± 8.618^a^	337 ± 11.563^ab^	0.070
**RDW (%)**	20.825 ± 1.996	19.85 ± 1.655	19.538 ± 1.088	0.276
**HDW (g/dL)**	22.7 ± 1.192^b^	27.013 ± 2.983^a^	25.643 ± 1.458^a^	0.001
**MPV (fL)**	7.95 ± 1.06	8.175 ± 0.767	8.225 ± 1.025	0.831
**NEUT (10e^9^/L)**	0.312 ± 0.056	0.304 ± 0.044	0.33 ± 0.045	0.558
**LYMPH (10e^9^/L)**	4.173 ± 0.488	4.37 ± 0.67	4.396 ± 0.482	0.682
**MONO (10e^9^/L)**	3.144 ± 0.756^a^	1.874 ± 0.349^b^	2.884 ± 0.538^a^	0.001
**EOS (10e^9^/L)**	0.01 ± 0.000^b^	0.011 ± 0.001^b^	0.025 ± 0.004^a^	<0.001
**BASO (10e^9^/L)**	0.03 ± 0.005^b^	0.044 ± 0.011^a^	0.031 ± 0.008^b^	0.006
**LUC (10e^9^/L)**	0.371 ± 0.094^b^	0.488 ± 0.079^a^	0.406 ± 0.071^ab^	0.029
**PDW (%)**	50.513 ± 13.542	48.05 ± 8.605	53.113 ± 12.004	0.686

WBC, white blood cell; RBC, red blood cell; HGB, haemoglobin; HCT, haematocrit; CH, cholesterol; MCV, mean corpuscular volume; CHCM, mean cell haemoglobin concentration; MCH, mean corpuscular haemoglobin; MCHC, mean corpuscular hemoglobin concentration; RDW, red blood cell distribution width; HDW, hemoglobin distribution width; MPV, mean platelet volume; NEUT, neutrophils; LYMPH, lymphocyte; MONO, monocytic cell count; EOS, eosinophil; BASO, basophil; LUC, large unstained cells; PDW, platelet distribution width. Data are presented as mean ± SEM (n = 8). ^a,b,c^ means with different superscript letters are significantly different (p < 0.05).

### BC supplementation significantly reduced the intestinal structural damage in piglets caused by ETEC K88 infection

**Figure 2:**
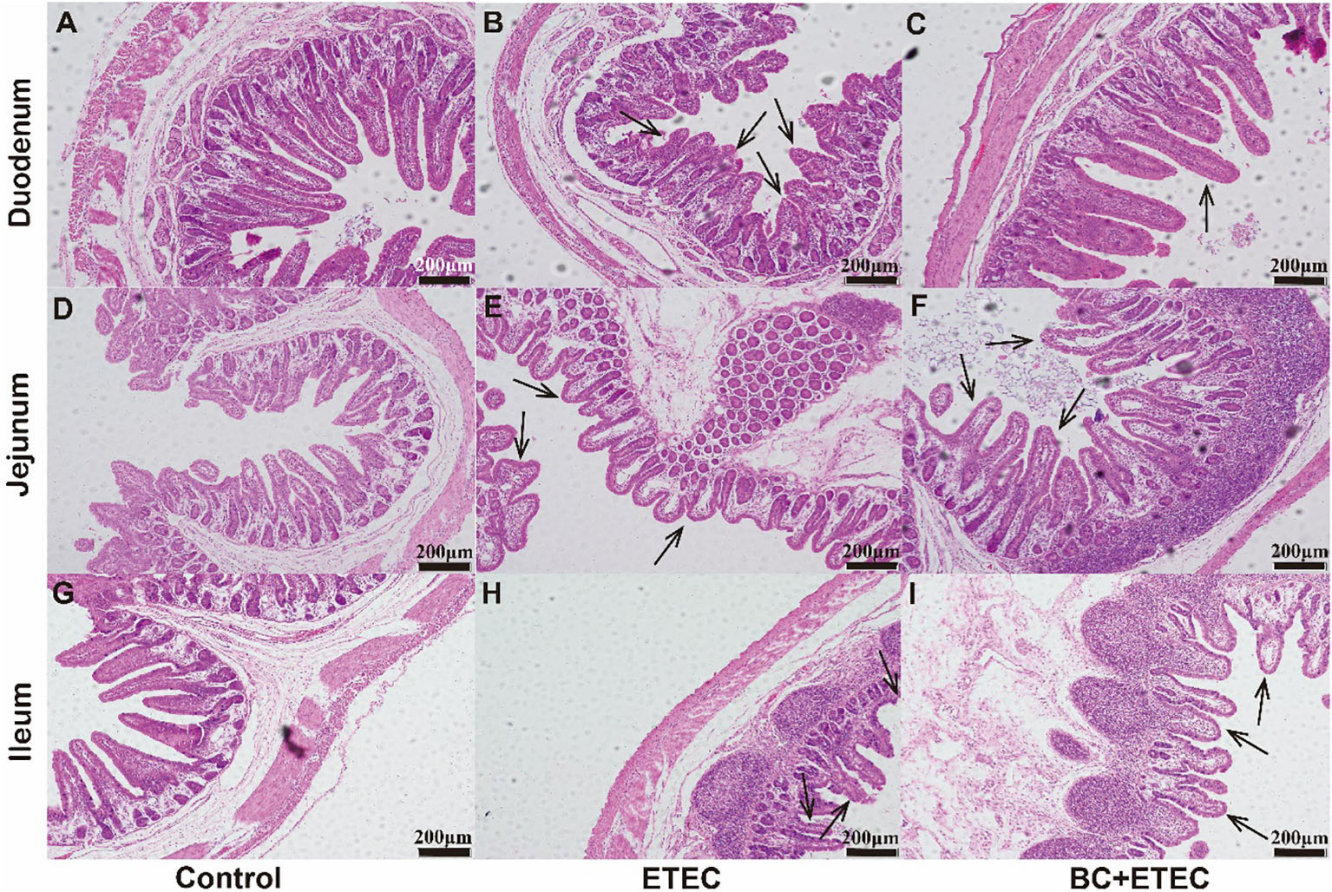
The effects of BC supplementation on intestinal damage in ETEC K88-infected piglets. **(A–I)** present H&E stained duodenum, jejunum, and ileum tissues. Arrows indicate intestinal villi. H&E: hematoxylin and eosin stain; Scale bar: 200 mm (H&E).

Further results demonstrated that ETEC infection caused severe damage to the intestinal villi, resulting in their shrinkage and even shedding (**Figures 2B, E, H**) when compared to the control group (**Figures 2A, D, G**). The BC supplementation significantly reduced the damage to the intestinal villi in piglets infected with ETEC K88 (**Figures 2C, F, I**) when compared to the ETEC group.

**Figure 3:**
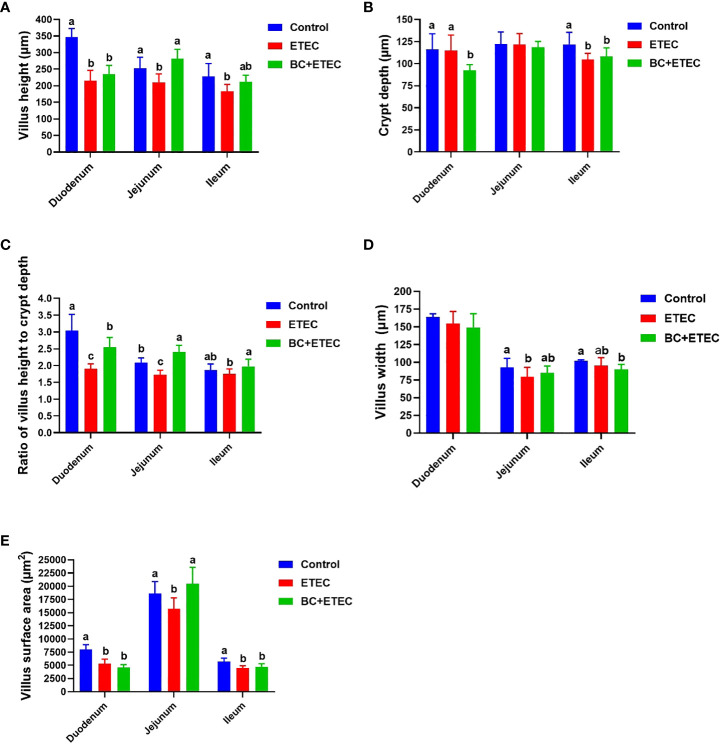
The effect of BC supplementation on intestinal structural damage in ETEC K88-infected piglets. **(A)** Villus weight. **(B)** Crypt depth. **(C)** Ratio of villus height to crypt depth. **(D)** Villus width. **(E)** Villus surface area. Data was presented as mean ± SEM (n = 8). ^a,b,c^ Means followed by different letters above the graphs indicate statistically significant differences (*p* < 0.05).

Compared to the control group, the duodenum showed a significant reduction in villus height (**Figure 3A**), the ratio of villus height to crypt depth (**Figure 3B**) and villus surface area (**Figure 3E**). The jejunum also exhibited a decrease in villus height (**Figure 3A**), the ratio of villus height to crypt depth (**Figure 3C**), villus width (**Figure 3D**) and villus surface area (**Figure 3E**). Additionally, the ileum displayed a decrease in villus height (**Figure 3A**), crypt depth (**Figure 3B**) and villus surface area (**Figure 3E**) in piglets from the ETEC group. However, piglets in the BC+ETEC group, which had received BC supplementation, showed a significant increase in villus height (**Figure 3A**), the ratio of villus height to crypt depth (**Figure 3C**) in the duodenum, jejunum and ileum and villus surface area (**Figure 3E**) in the jejunum.

### BC supplementation improved the antioxidant capacity of piglets infected with ETEC K88

**Figure 4:**
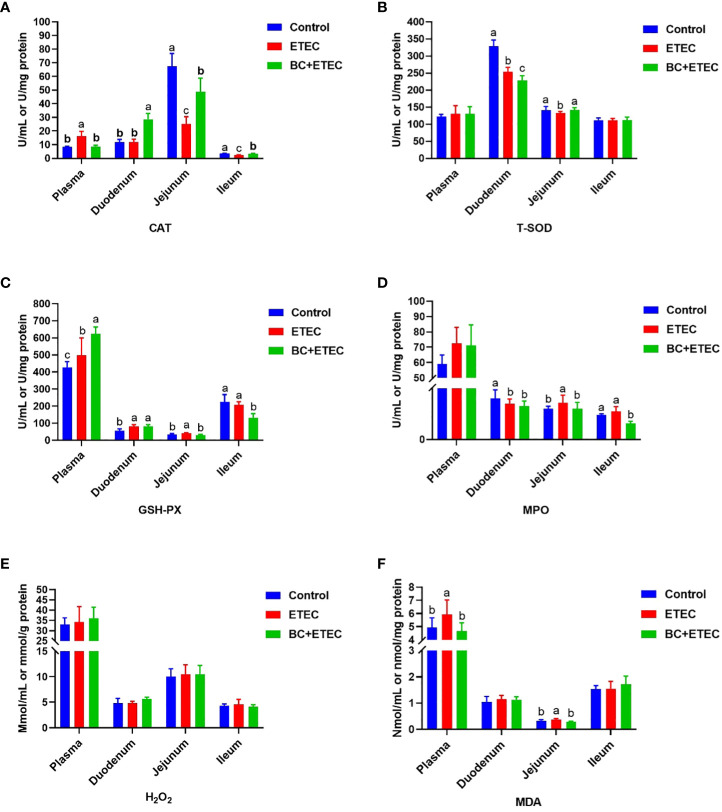
The effect of BC supplementation on intestinal antioxidant capacity in ETEC K88-infected piglets. **(A)** CAT activity. **(B)** T-SOD activity. **(C)** GSH-Px activity. **(D)** MPO activity. **(E)** H_2_O_2_ content. **(F)** MDA content. Data was presented as mean ± SEM (n = 8). ^a,b,c^ Means followed by different letters above the graphs indicate statistically significant differences (*p* < 0.05). CAT, Catalase; T-SOD, Total Superoxide Dismutase; GSH-Px, Glutathione peroxidase; MPO, Myeloperoxidase; H_2_O_2_, Hydrogen peroxide; MDA, Malondialdehyde.

Regarding the plasma, significantly higher activities of CAT and GSH-Px and the content of MDA were observed in the ETEC-infected animals as compared to the control and BC-supplemented groups (**Figures 4A, C, F**). For the duodenum, a significantly higher activity of GSH-Px but lower activities of T-SOD and MPO were observed in the ETEC-infected animals as compared to the control and BC-supplemented groups (**Figures 4B–D**). For the jejunum, significantly higher activities of GSH-Px and MPO as well as higher MDA contents were observed, along with lower activities of CAT and T-SOD in the ETEC-infected animals as compared to the control and BC-supplemented groups (**Figures 4A–D, F**). For the ileum, a significantly higher the activity of MPO but a lower CAT activity was observed in the ETEC-infected animals as compared to the control and BC-supplemented groups (**Figures 4A, D**). The content of H_2_O_2_ remained consistent across all animal groups.

### BC supplementation significantly decreased the intestinal epithelial apoptosis in ETEC K88-infected piglets

**Figure 5:**
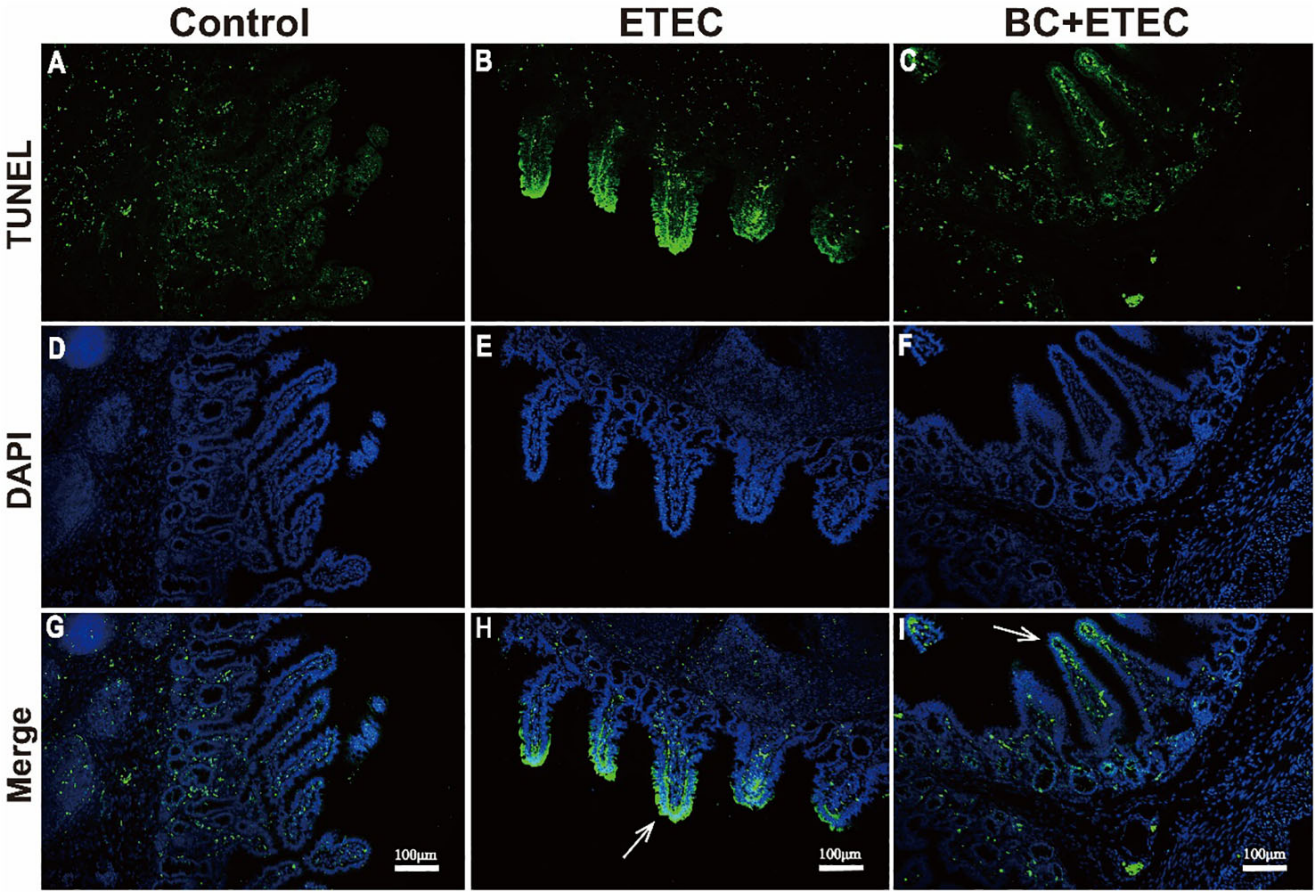
The effect of BC supplementation on intestinal epithelial apoptosis in ETEC K88-infected piglets. **(A, D, G)** control; **(B, E, H)** ETEC; **(C, F, I)** BC+ETEC. GTUNEL=terminal deoxynucleotidyl transferase dUTP nick end labeling; DAPI=4,6-diamidino-2-phenylindole. Arrows reflect the level of intestinal epithelial apoptosis. Scale bar: 100mm.

Compared to the control group (**Figures 5A, D, G**), the apoptosis of intestinal epithelial cells in piglets infected with ETEC K88 was enhanced (**Figures 5B, E, H**). However, when compared to the ETEC group, BC supplementation significantly inhibited the apoptosis of intestinal epithelial cells in infected piglets (**Figures 5C, F, I**).

### BC supplementation improved the gut microbial structure in young piglets infected with ETEC K88

Compared to the control group, the numbers of *Enterococcus*, *Clostridium*, and *Lactobacillus* in the jejunum, *Enterococcus* and *Clostridium* in the ileum, and *Escherichia coli*, *Bifidobacterium* and *Lactobacillus* in the colon of infected piglets decreased significantly. However, the numbers of *Escherichia coli* in the jejunum, total eubacteria in the ileum and *Enterococcus* and total eubacteria in the colon increased significantly (**Table 4**). Compared to the ETEC group, BC supplementation significantly increased the numbers of *Enterococcus*, *Clostridium* and *Lactobacillus* in the jejunum, *Enterococcus* and *Clostridium* in the ileum and *Escherichia coli*, *Bifidobacterium*, *Enterococcus* and *Lactobacillus* in the colon. However, it significantly decreased the number of total eubacteria in the colon in the BC+ETEC group. Although ETEC K88 infection did not affect the numbers of *Bifidobacterium* and total eubacteria in the jejunum, BC supplementation significantly increased their numbers in the BC+ETEC group. Additionally, BC supplementation tended to decrease the increase in the number of *E. coli* in the jejunum in piglets infected with ETEC K88 (**Table 4**).

**Table 4:** Effects of *B. coagulans* on intestinal microflora of young piglets infected with ETEC K88.

Item	Control	ETEC	BC+ETEC	*p*
Jejunum				
*Escherichia coli*	181.313 ± 19.303^b^	212.464 ± 9.866^a^	187.143 ± 22.627^b^	0.006
*Bifidobacterium*	262.65 ± 13.048	263.095 ± 13.166	243.65 ± 28.319	0.099
*Enterococcus*	162.587 ± 10.245^a^	114.48 ± 6.272^b^	178.718 ± 18.688^a^	<0.001
*Clostridium*	247.582 ± 23.834^a^	65.997 ± 6.597^c^	216.597 ± 23.339^b^	<0.001
*Lactobacillus*	349.943 ± 39.674^a^	287.314 ± 42.989^b^	349.702 ± 56.733^a^	0.020
Total eubacteria	7900 ± 1186.399^a^	8047.133 ± 951.228^b^	11238.367 ± 1070.617^b^	<0.001
Ileum				
*Escherichia coli*	117.143 ± 16.117^b^	121.813 ± 5.925^b^	147.644 ± 16.663^a^	0.009
*Enterococcus*	83.274 ± 13.176^a^	9.887 ± 1.209^b^	19.554 ± 2.726^b^	<0.001
*Clostridium*	0.024 ± 0.004^b^	0.013 ± 0.002^c^	0.075 ± 0.01^a^	<0.001
*Lactobacillus*	329.696 ± 52.68	316.159 ± 52.033	344.95 ± 61.577	0.721
Total eubacteria	7286.8 ± 1355.554^b^	11498.216 ± 1983.958^a^	10099.257 ± 1785.262^a^	0.007
Colon				
*Escherichia coli*	77.914 ± 2.965^a^	33.671 ± 3.972^c^	61.883 ± 7.546^b^	<0.001
*Bifidobacterium*	71.067 ± 9.035^a^	50.154 ± 8.654^b^	78.051 ± 10.623^a^	<0.001
*Enterococcus*	0.404 ± 0.045^c^	0.614 ± 0.211^b^	0.895 ± 0.107^a^	<0.001
*Clostridium*	147.75 ± 17.479	138.162 ± 17.313	154.859 ± 17.938	0.187
*Lactobacillus*	213.657 ± 14.179^a^	69.616 ± 13.463^b^	220.143 ± 32.852^a^	<0.001
Total eubacteria	10567.5 ± 2341.378^b^	12944.762 ± 1188.861^a^	9405.714 ± 228.714^b^	<0.001

Data was presented as mean ± SEM (Jejunum and colon n = 8; Ileum n=5). ^a,b,c^ means with different superscript letters are significantly different (p < 0.05).

## Discussion

Previous studies have suggested that probiotics can be used as feed additives to enhance the growth performance of weaned piglets. This is achieved by reducing intestinal injury and regulating the gut microbiota (Bermudez-Brito et al., 2012; Biswas et al., 2022). *Bacillus coagulans* exhibits biological activity in the gastrointestinal tract; it can improve the intestinal structure and function and regulate the gut microbiota (Konuray and Erginkaya, 2018; Lavrentev et al., 2021). The underlying mechanism is that *B. coagulans* not only regulates host symbiotic microbiota by inhibiting the proliferation of pathogenic microorganisms but also improves the host immune system by normalising both the quantitative parameters of the immune system and immune cells functional activity (Cao et al., 2020). However, the effects of BC on the intestinal structure, function and gut microbiota in ETEC K88-infected young piglets are still unclear. In this study, our findings are the first to indicate that BC supplementation improved ADFI, intestinal morphology, intestinal epithelial cell apoptosis, redox imbalance and gut microbial dysbiosis in ETEC K88-infected young piglets.

Frequently, ETEC K88 infection leads to watery diarrhoea and reduced food intake; long-term diarrhoea can result in intestinal injury in piglets aged 7-15 days (Xia et al., 2015; Sun and Kim, 2017). In this study, compared with the ETEC group, although the faecal score of piglets in the BC+ETEC group was not significant, there was a downward trend (*p* > 0.05) (data no shown). Furthermore, the increased ADFI (**Figure 1**) provides additional evidence of the positive effects of BC supplementation on intestinal functions in ETEC K88-infected young piglets. Compared with the control group, ETEC K88 infection appeared to hinder the growth of piglets, whereas BC supplementation tended to increase the BW of ETEC K88-infected piglets by Day 7 of the trial (**Figure 1**). These results suggest that BC can alleviate the decline in ADFI caused by ETEC K88 infection in young piglets.

Previous studies have reported that ETEC infection significantly affected blood biochemical and haematological parameters in piglets (Garas et al., 2017; Le Devendec et al., 2018). Abnormal changes in these indicators suggest that ETEC infection damaged not only the intestine but also other tissues and organs. Consistent with a previous study, our results reveal that ETEC K88 infection significantly reduced the BUN level, which can be associated with renal function (Zhang et al., 2023). BC supplementation alleviated the decrease in BUN level in ETEC K88-infected piglets (**Table 2**), suggesting that BC can alleviate renal dysfunction caused by ETEC K88 challenge in piglets. In this study, ETEC K88 infection also significantly reduced the WBC and MONO, which can be associated with the host immune level (Zhang et al., 2022; Zhang et al., 2023). This study showed that BC supplementation alleviated the decrease in WBC and MONO in ETEC K88-infected piglets (**Table 3**), suggesting that BC can alleviate decreased immune levels caused by ETEC K88 challenge in piglets. Moreover, BC supplementation did not exacerbate the abnormal changes in biochemical and haematological parameters in young piglets resulting from ETEC K88 challenge. This leads us to infer that BC can be used as a potential nutrient regulator to prevent ETEC K88 infection in young piglets.

Generally, ETEC K88 infection in young piglets is associated with severe intestinal damage and dysbiosis of the gut microbiota (Xia et al., 2015; Zhang et al., 2022). Plasma D-xylose is useful markers and reflects the integrity of the intestinal structure (Hou et al., 2011; Zhang et al., 2019). In this study, BC supplementation reduced the damage to the intestine in ETEC K88-infected young piglets, as indicated by an increase in the content of plasma D-xylose (**Table 2**). Moreover, BC supplementation mitigated the intestinal damage of ETEC K88-infeced piglets by improving villus height, the villus height-to-crypt-depth ratio of the duodenum, jejunum and ileum, as well as the villus surface area of the jejunum (**Figures 2**, **3**). These results suggest that BC supplementation alleviated the decline in ADFI by reducing the ETEC K88-induced intestinal injury in ETEC K88-infected young piglets.

Previous studies have reported that ETEC K88 can lead to intestinal damage by causing excessive stress repression, including immune stress and oxidative stress in the intestine of piglets (Jiménez et al., 2020; Kim et al., 2022). To further reveal the relevant mechanism of BC supplementation in alleviating intestinal injury in ETEC K88-infected young piglets, this study determined the intestinal morphology and redox level of ETEC K88-infected piglets. Oxidative stress caused by ETEC K88 infection is the main cause of intestinal injury in piglets (Pu et al., 2020; Sun et al., 2022). In this study, BC supplementation increased the antioxidant capacity of the intestine in ETEC K88-infected piglets, as indicated by an increase in the antioxidant index levels of CAT, T-SOD and GSH-Px and a decrease in MPO and the oxidative metabolite MDA in the jejunum. In addition to the above results and previous reports that dietary BC supplementation reduces intestinal damage by increasing the antioxidant capacity in weaned piglets and growing-finishing pigs (Fu et al., 2019; Sun et al., 2022), our results also indicate a new mechanism of BC reducing ETEC K88-induced intestinal injury in young piglets. The apoptosis of intestinal epithelial cells is essential for the renewal of intestinal cells and the maintenance of normal intestinal structure and function. An imbalance between apoptosis and cell regeneration can seriously damage intestinal structure and function (Zhu et al., 2014). In this study, ETEC K88 challenge promoted the apoptosis of intestinal epithelial cells in young piglets. However, BC supplementation reduced intestinal epithelial cell apoptosis in ETEC K88-infected young piglets. These results suggested that BC supplementation reduced ETEC K88-induced intestinal injury by increasing the intestinal antioxidant capacity and improving the imbalance between apoptosis and cell regeneration in ETEC K88-infected young piglets.

Although infancy is crucial for the establishment of gut microbiota in piglets (Han et al., 2019), the gut microbial structure in infancy is unstable and easily influenced by the surrounding environment (Xu et al., 2016). An unstable gut microbial community is one of the main factors causing diarrhoea in suckling piglets (Bauer et al., 2006). Therefore, reducing or avoiding the influence of external conditions on the gut microbial composition of suckling piglets is crucial for the gut microflora and health of pigs. In a previous study, BC improved the growth performance and diarrhoea index by altering the gut microbial composition in weaned piglets (Sun et al., 2022). In this study, compared with the control group, ETEC K88 challenge significantly increased the numbers of *Escherichia coli* and total eubacteria in the jejunum, total eubacteria in the ileum and *Enterococcus* and total eubacteria in the colon. However, ETEC K88 challenge significantly reduced the numbers of *Enterococcus, Clostridium and Lactobacillus* in the jejunum, *Enterococcus* and *Clostridium* in the ileum and *Escherichia coli*, *Bifidobacterium* and *Lactobacillus* in the colon in ETEC K88-induced piglets. These results suggest that ETEC K88 challenge caused a significant imbalance of the gut microbiota. However, BC supplementation reversed the ETEC K88-induced increase in the numbers of *Escherichia coli* in the jejunum and total eubacteria in the colon and the decrease in the numbers of *Clostridium, Enterococcus and Lactobacillus* in the jejunum and *Escherichia coli* and *Bifidobacterium* and *Lactobacillus* in the colon. These results suggest that BC supplementation significantly alleviated the effects of ETEC K88 infection on the gut microbiota structure of the jejunum and colon in ETEC K88-infected piglets. Over, our results indicate that BC could regulate the gut microbial composition by alleviating ETEC K88-induced gut microbial imbalance in young piglets (see also Bauer et al., 2006; Hu et al., 2016; Xu et al., 2016; Wang et al., 2017; Han et al., 2019; Patil et al., 2020; Luo et al., 2022; Sun et al., 2022).

## Conclusions

In conclusion, BC supplementation could prevent the decline in average daily feed intake in ETEC K88-infected piglets by attenuating intestinal injury and regulating gut microbiota. Our research provides a reliable theoretical basis for the application of BC in the swine industry.

## Data availability statement

The raw data supporting the conclusions of this article will be made available by the authors, without undue reservation.

## Ethics statement

All experimental procedures were approved by the Institutional Animal Care and Use Committee at Wuhan Polytechnic University (approval code WPU201910001). The study was conducted in accordance with the local legislation and institutional requirements.

## Author contributions

YZ: Formal Analysis, Writing – original draft, Writing – review & editing. XT: Formal Analysis, Writing – review & editing, Methodology. YD: Data curation, Formal Analysis, Investigation, Resources, Writing – review & editing. RL: Formal Analysis, Writing – review & editing, Data curation. MS: Writing – review & editing, Data curation, Formal Analysis, Software. DY: Writing – review & editing, Conceptualization, Resources. TW: Data curation, Methodology, Writing – review & editing, Project administration. LW: Data curation, Writing – review & editing, Methodology, Validation. DZ: Writing – review & editing, Data curation, Investigation. YH: Conceptualization, Funding acquisition, Resources, Writing – original draft, Writing – review & editing.
